# Comparison of Catastrophic Out-of-Pocket Medical Expenditures in the US and South Korea

**DOI:** 10.1093/geroni/igab046.3028

**Published:** 2021-12-17

**Authors:** Narae Kim, Mireille Jacobson

**Affiliations:** University of Southern California, Los Angeles, California, United States

## Abstract

To date, relatively few studies have examined catastrophic out-of-pocket medical spending in the United States, especially in comparison to other high-income countries. We compared catastrophic out-of-pocket medical spending among adults age 65 and older in the United States versus South Korea, a high-income country with national health insurance that is often overlooked in cross-country comparisons. We defined catastrophic medical spending as health care expenditure for the past two years that exceeds 50% of one’s annual household income. Using data from the 2016 Health and Retirement Study (HRS) and Korean Longitudinal Study of Aging (KLoSA), we performed a logistic regression to examine the factors affecting catastrophic out-of-pocket medical spending for older adults in both countries. We also performed a Blinder-Oaxaca decomposition to compare the contribution of demographics factors versus health system-level factors to catastrophic out-of-pocket medical spending. The proportion of respondents with catastrophic out-of-pocket medical expenditure was higher in the US; the proportion was 5.8% and 3.0% in the US and South Korea, respectively. Both in the US and South Korea, respondents who were in the lower-income quartiles, who had experienced a stroke or had diabetes, and who rated their health as poor had higher odds of catastrophic out-of-pocket medical expenditure. The Blinder-Oaxaca non-linear decomposition showed that the significant difference in the rate of catastrophic out-of-pocket medical spending between the two countries was attributable to unobservable system-level factors, not observed differences in the sociodemographic characteristics between the two countries.

